# Expanding the biomass resource: sustainable oil production via fast pyrolysis of low input high diversity biomass and the potential integration of thermochemical and biological conversion routes

**DOI:** 10.1016/j.apenergy.2016.05.088

**Published:** 2016-09-01

**Authors:** J. Corton, I.S. Donnison, M. Patel, L. Bühle, E. Hodgson, M. Wachendorf, A. Bridgwater, G. Allison, M.D. Fraser

**Affiliations:** a Institute of Biological Environmental and Rural Sciences, Aberystwyth University, Gogerddan, Aberystwyth, Ceredigion SY23 3EB, UK; b European Bioenergy Research Institute, Aston University, Birmingham B4 7ET, UK; c Department of Grassland Science and Renewable Plant Resources, University of Kassel, Steinstr. 19, 37213 Witzenhausen, Germany

**Keywords:** Integrated processing, Conservation biomass, Fast pyrolysis, Biomass availability, Low input high diversity, Biocrude

## Abstract

Waste biomass is generated during the conservation management of semi-natural habitats, and represents an unused resource and potential bioenergy feedstock that does not compete with food production. Thermogravimetric analysis was used to characterise a representative range of biomass generated during conservation management in Wales. Of the biomass types assessed, those dominated by rush (*Juncus effuses*) and bracken (*Pteridium aquilinum*) exhibited the highest and lowest volatile compositions respectively and were selected for bench scale conversion via fast pyrolysis. Each biomass type was ensiled and a sub-sample of silage was washed and pressed. Demineralization of conservation biomass through washing and pressing was associated with higher oil yields following fast pyrolysis. The oil yields were within the published range established for the dedicated energy crops miscanthus and willow. In order to examine the potential a multiple output energy system was developed with gross power production estimates following valorisation of the press fluid, char and oil. If used in multi fuel industrial burners the char and oil alone would displace 3.9 × 10^5^ tonnes per year of No. 2 light oil using Welsh biomass from conservation management. Bioenergy and product development using these feedstocks could simultaneously support biodiversity management and displace fossil fuels, thereby reducing GHG emissions. Gross power generation predictions show good potential.

## Highlights

• Biomass is generated during management of low input high diversity (LIHD) landscapes.
• Samples of LIHD biomass were subjected to fast pyrolysis.
• Demineralization through washing and pressing was associated with higher oil yields.
• Oil yields were within the range following fast pyrolysis of Miscanthus and Willow.
• Gross estimates of 4 × 10^5^ tonne per year of oil could be displaced using Welsh LIHD biomass.

## 1 Introduction

Declining fossil fuel reserves and the negative environmental impact associated with their use has driven research into alternative and sustainable alternatives. Renewable energy supplies in the form of liquid, gas and solid fuel are reliant on biomass as a feedstock [1]. Production of these products from biomass grown in areas that could be used to grow food has received criticism [2]. This has led to research into the use of low input high diversity (LIHD) biomass, that is generated during the management of semi-natural landscapes, for energy production [2], [3], [4], where there is no negative impact, either direct or indirect, on food production.

Across Europe semi-natural landscapes are in decline due to changes in agricultural practices [5]. For many semi-natural vegetation communities in the UK overstocking in the 1980s was followed by understocking in the late 1990’s and beyond. Both situations are detrimental to biodiversity [6]. One way to preserve the biodiversity of these habitats and landscapes is to cut and remove the biomass. If they are not managed by cutting landscape degradation takes place and biodiversity decreases as dominant plant species spread [7], [8]. Many conservation bodies and charities employ this management method and generate large amounts of biomass as a consequence [9]. Currently the biomass generated during conservation management is treated as waste and left for decomposition. However, this widespread and plentiful resource could be used as a feedstock for bioenergy, plus its use could have a positive impact upon national GHG targets, biodiversity and ecosystem services [3].

In 2006 Tilman et al. [2] highlighted the advantages of LIHD grasslands for biofuel production in the USA, and European perspectives have been provided [3], [10], [11], [12]. Work by Tonn et al. [13] employed biomass from land in Germany hat was no longer utilised for livestock production to make bioenergy in the form of combustion fuel. Fischer Tropsch fuel production was modelled by Corton et al. [3]. The potential of the Estonian resource has been reported Heinsoo et al. [10]. More recently Meerbeek et al. [14] examined conservation biomass alongside roadside verge waste as feedstocks for biogas production via anaerobic digestion (AD). However, none of these works examined process routes that utilise fast pyrolysis.

One process that was developed around the specific requirements (low sugar and high mineral content) of LIHD biomass was the Integrated Generation of Solid Fuel and Biogas from Biomass (IFBB) procedure [4], [15]. The IFBB process is a green biorefinery model [16] whereby hydrothermal pre-treatment and mechanical dehydration (HPMD) by pressing, using a screw press, produces a fibrous product (press cake) with a significantly lower mineral composition than that of the original feed-stock. The intention was to make a better combustion fuel through partial demineralisation [17]. Lower alkali metal composition is correlated with a higher ash softening temperature and this is desirable in a combustion fuel. Demineralisation also has positive implications for emissions and combustion chamber preservation by lowering sulphur (SO*_X_*) and chlorine concentrations [18]. In the IFBB process the fluid generated during pressing was used as a feedstock for anaerobic digestion and the biogas generated used as an energy source for the system, ensuring a favourable energy balance [15] . The volume of fluid generated is dependent upon the moisture content, water wash pre-treatment employed and species composition of the feedstock. An approximation for guidance would be ∼0.3 tonnes of fluid (wet weight) per tonne of dry feedstock [3].

One process route that could potentially be integrated into a green refinery system (such as IFBB) is fast pyrolysis. Fast pyrolysis is a process whereby organic vapours, gas, water and char are produced by heating biomass to 450–600 °C. The process is characterised by high heating rates and low residence times for the organic vapours that are generated [19]. The organic vapours and water are rapidly condensed to form a homogenous bio-oil, and this oil is the primary and major product of fast pyrolysis. The oil can be used as a fuel for burning in boilers following burner modification, steam reformed to make hydrogen fuel or processed to produce chemicals [20]. Fast pyrolysis is also ideal for decentralised systems as the liquid product has a very high energy density and can be readily transported to the point of use. In the current work we subjected biomass in the form of silage that had been hydrothermally pre-treated and screw-pressed (as in the IFBB process), plus control ensiled biomass that had not been pressed, to fast pyrolysis.

An analysis of the impact of semi-demineralisation upon the yields from fast pyrolysis was one aspect of the current study. Another aspect was to examine the broad potential of a system that exploits AD of the press fluid with fast pyrolysis of the press cake to create multiple energy carriers. Work by Lou et al. [21] demonstrated that de-ashing and mineral removal was important for successful bio-oil production using bamboo as a feedstock. The presence of minerals had an influence upon the bio-oil composition and encouraged undesirable CO_2_ production during fast pyrolysis. Following HPMD however, only partial demineralisation was achieved and we have yet to establish the effect of the process on product yields following fast pyrolysis. Mineral composition is known to be dependent on species and habitat origin as well as pre-treatment [22]. In this work we examined the impact of vegetation community type on the product yields and the characteristics of those products as obtained from fast pyrolysis of LIHD biomass.

Biomass harvested from conservation of semi natural habitats is heterogeneous being made up of a diverse range of plant species. In this paper we seek to determine the suitability of LIHD biomass as a feedstock for oil production via fast pyrolysis following conservation through ensiling and after a washing and pressing pre-treatment. The results of this conversion are compared to those generated from fast pyrolysis of the purpose grown bioenergy crops miscanthus and willow.

Combining thermochemical and biological conversion routes (fast pyrolysis and AD respectively in the current study) into a unified system has been examined previously but not in the manner proposed in this work. One processing system that combines thermochemical and biological conversion routes has been reported previously by Monlau et al. [23] and is a good example of how conversion routes can be cascaded to encourage positive energy balances. Monlau et al. [23] used the digestate waste produced during anaerobic digestion as a feedstock for pyrolysis, once dried by the excess energy from the anaerobic digestor. Therefore the system exploits a feedback between the two conversion routes (through drying) and this differs from the approach taken by Inyang et al. [24] for example, who pyrolysed digestate without integrating a feedback loop between the conversion routes.

In the final section a potential system is illustrated that combines anaerobic digestion of the press fluid alongside oil and char production via fast pyrolysis. Estimations of gross energy output are presented. This provides some perspective as to the scope and potential scale of the resource and system. It is envisaged that the gap between research and commercial realization of the fast pyrolysis conversion route may benefit from the energy generated from anaerobic digestion of press fluids, in combination with the valorisation of the char bi-product. Novel experimental data was generated by subjecting representative UK LIHD feedstocks to fast pyrolysis. Gross theoretical outputs of the whole system are calculated using published resources. Therefore this work implements a cascading processes promoted by the notion of industrial symbiosis [25] in order to increase the feasibility of biomass conversion.

## 2 Materials and methods

### 2.1 Experimental design

During research associated with the IFBB system six distinct UK semi-natural vegetation communities that were dominated by different plant species were harvested and prepared by (a) ensiling only, or (b) ensiling followed by hydrothermal pre-treatment with mechanical dehydration (HPMD; generating a press cake and a fluid). Each community type had three fenced established plots measuring 10 m by 10 m. Resource constraints meant that we prioritised our analyses on four fast pyrolysis runs which tested the impact of demineralisation by HPMD on the products of fast pyrolysis.

Two different types of semi-natural feedstock were processed (comparing the silage and press cake of each). Thermogravimetric analysis (TGA) was used to characterise the vegetation types. The two (out of six) biomass samples with the most distinct volatile solid compositions, rush and bracken, were then chosen for conversion via fast pyrolysis. In order to provide representative forms of LIHD biomass, feedstocks employed in the current study were mixed species but taken from areas dominated by particular plant species. The biomass was therefore representative of the biomass that would be cut by an environmental contractor whereby the whole sward would be harvested for processing and not individual species. Raw biomass (not ensiled) was not used for thermochemical experimentation in the current study because herbage preservation (in this case through ensiling) is generally accepted as important to commercial bioenergy generation management [26].

### 2.2 Harvesting

Biomass samples were obtained by harvesting randomly selected strips within each of three random quadrats measuring 10 m × 10 m per site. Harvesting was conducted using a finger-bar mower (Shank’s Pony GC135, Honda, Slough, U.K.). The cutting height was set to 5 cm from ground level. Harvesting took place during August. Six sites were harvested and two were selected for fast pyrolysis.

### 2.3 Silage production

Triplicate biomass samples from each site were chopped (separately) into approximately 5 cm lengths using a clean static forage chopper (Model BCS, 15kw; Electra, France). Approximately 25 kg of chopped biomass was then placed and consistently compacted into 60 l polyethylene barrels. Following a 4 month ensiling period biomass samples destined for fast pyrolysis were extracted from the centre of the barrels by hand.

### 2.4 Press cake production

Pre-treatments were conducted in the pilot scale ProGrass processing plant [27]. Processing occurred whilst the pilot plant was based at The Institute for Biological Environmental and Rural Sciences (IBERS) at Aberystwyth University (UK). Approximately 20 kg of silage from each replicate sub plot from each site was hydrothermally conditioned using sprinklers over biomass placed on a moving conveyor for 20 min using fresh tap water pre-heated to a temperature of 25 °C. Following hydrothermal pre-treatment the silage was pressed using an AV screw press (Anhydro Ltd., Kassel, Germany). The screw press had a perforation of 1.5 mm; it was a conical screw type (pitch 1:6) with a screw rotation speed of 3 rev min^−1^ [28]. Following pressing the fibrous press cake (PC) was collected.

### 2.5 Sample screening using Thermogravimetric analysis (TGA)

Triplicate silage and press cake samples from the triplicate plots on each of the six sites (Table 1) were independently weighed then dried to constant weight for 52 h at 60 °C. The samples were separately milled through a 1 mm hammer mill (Retsch, Germany). The milled materials were sieved with an electric vibrating sieve to a particle size of between 0.25 mm and 1 mm.

A Perkin Elmer Pyris 1 thermogravimetric analyser (Shelton, U.S.A.) was used to conduct the TGA experiments. The ambient gas was nitrogen (N_2_) with a flow rate of 20 ml min^−1^. The following program was used: heat from 32–105 °C at 25 °C min^−1^; hold at 105 °C for 5 min; heat from 105–905 °C at 25 °C min^−1^; hold at 905 °C 15 min; cool from 905–200 °C in at 25 °C min^−1^; heat from 200–575 °C in air with a flow rate of 20 ml min^−1^ at 100 °C min^−1^; hold at 575 °C for 15 min.

**Fig. 1:**
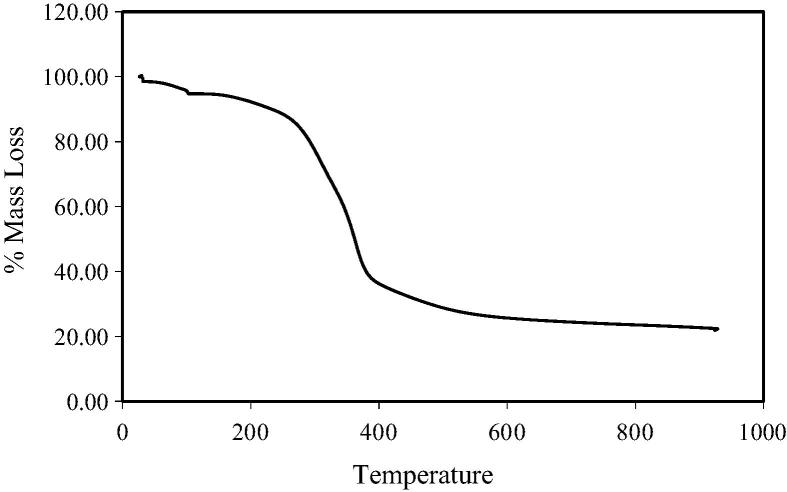
An example thermogravimetric mass loss curve, the mass losses within particular thermal boundaries are used to establish the mass of specific component groups.

The mass of the moisture and volatile fractions was established by calculating the mass loss during specific temperature ranges: moisture content (MC) 32–105 °C; volatile content (VC) 105–550 °C. The ash composition was the mass remaining following the final components of the TGA sequence when air was utilised as an ambient gas to enable combustion, leaving only the inorganic ash fraction. The fixed carbon (FC) fraction was calculated using the equation FC = 100 − MC − VC − ash. The FC and VC were expressed as DM ash free. A TGA mass loss curve is presented in Fig. 1.

The volatile component of a feedstock is composed of organic compounds that evaporate or sublimate from the feedstock at temperature ranges between 105–550 °C these generally condense to form the oil produced in a fast pyrolysis system.

### 2.6 Bulk density measurements

Biomass samples weighing 100 g were placed into a graduated 250 ml glass cylinder and tapped down by hand. The volume (*V*°) was recorded. To calculate the bulk density in g per ml the formula *m*/*V*° = bulk density (g/ml) was employed (*m* = mass in g).

### 2.7 Dry matter determination and sample preparation for fast pyrolysis processing

Triplicate samples of silage and press cake from the two chosen sites were independently weighed then dried to a constant weight for 52 h at 60 °C in an air circulated oven and then reweighed in order to establish the DM content. This drying method was implemented to ensure minimal volatile solids loss and is not anticipated that this method would be used at commercial scale. At scale, if pressed biomass is suitable for pyrolysis, then driers powered by biogas generated from press fluid digestion would be implemented. The viability of this method has been examined [15] as applied to drying the press cake for combustion fuel production. Anaerobic digestion of the press fluids was optimized and reported in Corton et al. [29].

The triplicate silage and press cake sub-samples were bulked together separately by combining equal masses of each and by mixing by hand. Following homogenisation and bulking of the samples, sub-samples of the bulked press cake and silage samples from the two sites were milled through a 1 mm hammer mill (Retsch, Germany). The milled materials were remixed again and milled again to ensure homogeneity before been sieved to a particle size of 0.25 mm to 1 mm. Four bulked samples were therefore produced for thermochemical conversion: press cake and silage biomass from each of the TGA selected sites.

### 2.8 Fast pyrolysis apparatus and experimental procedure

**Fig. 2:**
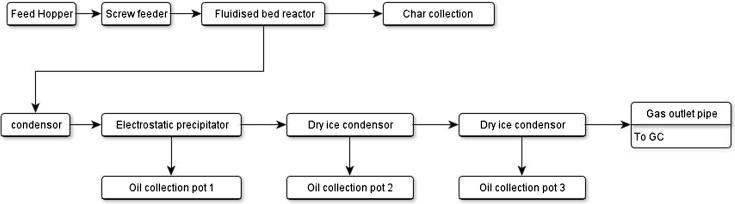
A process chart of the bench-scale fast pyrolysis set up and utilised in the current study.

A bench scale fast pyrolysis set up was employed with a 300 g per h^−1^ feedstock input rate. A process chart of the rig set up is shown in Fig. 2.

The fluidised bed medium was composed of 150 g of quartz sand with a particle size between 500–600 μm. The fluidising rate was established after considering the bulk densities of the feedstock. The bulk densities (Table 2) are within the range between bagasse (159 kg m^−3^) and miscanthus (274 kg m^−3^) both of which have successfully been pyrolysed with a fluidising N_2_ flow rate of 12 L min^−1^ and this rate was therefore employed.

In order to obtain a mass balance, the following rig components were weighed before and after pyrolysis: glass transition pipe between the condenser and the cyclone; the condenser; the glass transition pipe between the condenser and the electrostatic precipitator; the electrostatic precipitator (ESP); three oil pots; and the gas outlet pipe.

Prior to experimentation the reactor was insulated, water was used to cool the condenser and the dry ice condensers were filled with frozen CO_2_ and topped up with acetone. Acetone was intermittently added during pyrolysis in order to keep the condensers full. As a consequence a temperature of approx. −30 °C was maintained in the two condensers.

The volume of gas (non-condensable fraction) that was produced was measured through the gas outlet pipe using a gas meter. Gas composition was established using inline gas chromatography (GC). GC analysis began 20 min before biomass was fed into the reactor sampling every three minutes.

Once the fluidised bed was at a temperature of 500 °C and the reactor’s continuous feed system was started processing began. A thermocouple was used to monitor the fluidised bed temperature. Biomass was fed into the reactor through a N_2_ flushed feed pipe. The N_2_ was fed into the top of the biomass feeder unit. The biomass feeding mechanism was a screw auger. The pyrolysis run duration time was 1.5 h.

During pyrolysis the temperature of the fluidised bed was monitored. No significant fluctuations occurred on the experimental runs. The feedstock was delivered into the centre of the fluidised bed reactor and following pyrolysis the products entered a cyclone unit. The char was separated from the product mix by the cyclone and collected in a char pot beneath the reactor. The product mix then passed through a transition pipe to the condensing system.

Some pyrolysis vapours were condensed in the water cooled condenser and collected in oil pot 1. Uncondensed vapours and remaining aerosols then entered the electrostatic precipitator and were precipitated. These products were also collected in oil pot 1. Further remaining uncondensed vapours were condensed using the dry ice condensers and the oil was distributed into oil pots 2 and 3 (Fig. 2). Any remaining uncondensed volatiles were filtered out of the system by a cotton wool filter. Gases that were none condensable passed through the filter and into the GC.

### 2.9 Compositional analysis

The C, H, N and S compositions of the bulked samples were established using a FlashEA® 1112 Elemental Analyzer (MEDAC Ltd, Surrey, UK). All the tests were duplicated and means were calculated. Oxygen composition was calculated by difference (100 − (Ash + C + H + N) = O).

The total ash content of the samples was determined by weighing the sample before and after incineration in a muffle furnace at 550 °C for 5 h. It is the component remaining after the combustible material has been burned off.

Higher heating values (HHVs) were established using CHNO composition and employing the use of Friedl’s equation [30]: HHV = 0.0355 × C2 − 23.2 × C − 223 × H + 0.512 × C × H + 13.1 × N + 20,600 (kJ kg^−1^ DM). Lower heating values (LHVs) were calculated using: LHV^dry^ (MJ kg^−1^) = HHV^dry^ − 2.442 × (8.936H–100) [31].

Neutral-detergent fibre (NDF) was determined using the Gerhardt fibrecap system [32] as developed from the Van Soest procedure [33]. NDF is the fibre fraction regarded as cell wall and is the residue, corrected for ash, after refluxing the sample for 1 h in a neutral detergent solution. Acid-detergent fibre (ADF) represents the lignin and cellulose fractions of the plant cell wall. ADF was determined using the Gerhardt fibrecap system also developed from the Van Soest procedure [33]. ADF is defined as the loss on ignition of the dried residue remaining after digestion with an acid detergent solution. Acid detergent lignin (ADL) was determined gravimetrically. Initially the acid detergent residue was obtained, it was then treated with 72 % sulphuric acid to solubilise the cellulose and isolate crude lignin plus ash. The cellulose and hemicellulose content of the biomass samples were calculated from the ADL, ADF and NDF analyses. Given that ADF consists of cellulose and lignin, and NDF comprises cellulose, hemicellulose and lignin, the cellulose and hemicellulose content of samples were calculated by the equations: cellulose = ADF − ADL; hemicellulose = NDF – ADF [33]. The composition of the following minerals was established: S, K, Mg, Ca, P and Na as reported by [28].

The water content of the liquid component in the fast pyrolysis product stream was determined using the Karl Fischer (KF) titration method. A mean from three replicate measurements was calculated. The KF moisture content was measured using a Mettler Toledo V20 Volumetric Karl Fischer Titrator (Mettler Toledo, U.S.A.). The figures represent percentages of the total product stream. Distilled water was used to calibrate the instrument before the analysis of each oil sample. The reaction water generated during processing was calculated as the difference between the KF moisture content of the liquid product fraction and the feedstock moisture content.

The derivatized oil and aqueous fractions were chemically profiled by gas chromatography mass spectroscopy (GC–MS) after derivatization. Trimethylsilyl (TMS) derivatization was employed on the basis that it is effective with the majority of polar chemicals [34]. Derivatization masked polar moieties, increased volatility and allowed effective chromatography on a non-polar GC column at high temperature. Derivatized oil and aqueous samples were subjected to GC–MS analysis using an Agilent 6890 series GC, a 5973 series mass sensitive detector and a 7683 autosampler (Agilent, U.S.A). The column used was a Varian VF-5MS (Palo Alto, California USA) column with a length of 30 m; the inside diameter was 0.25 mm and the film thickness was 0.25 μm. The method used was as follows: injection volume of 1 μl; inlet split of 40:1; inlet temperature of 280 °C; flowrate of 1 ml min^−1^. Oven parameters were 80 °C for 1 min, heat at 20 °C min^−1^ to 280 °C, heat at 50 °C min^−1^ to 330 °C. Mass spectrometer parameters were scan limits of 5*m*/*z* to 50*m*/*z* (3.25 scans s^−1^) and a solvent delay of 3 min was employed between injection and scan start.

### 2.10 Statistical analysis

Data derived from the TGA of biomass samples were analysed using an analysis of variance (ANOVA) with a post hoc multiple comparisons test (Student Neuman Keuls). Genstat [35] statistical software was used for the ANOVA.

### 2.11 Comparative analysis

In order to obtain a perspective of how LIHD biomass compares to the purpose grown biofuel crops Miscanthus and Willow, data was compared to that from Greenhalf et al. [36] which used the same methods in the same laboratory.

### 2.12 Gross power production estimates

Bio-oil and char combustion was modelled utilising burners that are adaptable to a variety of fuel types. Both char and bio oil have been used for prolonged periods in industrial burners [20]. No char densification (pelleting for example) is factored in as a consequence. Oil and char production levels are based on the mean oil and char yield results following fast pyrolysis of press cakes generated from LIHD press cake biomass (Table 8). Feedstock availability is based on Corton et al. 2013 [3]. The press fluid production estimates and consequential methane production are taken from work associated with Corton et al. [29] and include concentrating the fluid prior to digestion by utilising a settling tank and digesting the concentrate. The gross energy estimates are reported in Table 8 employing the system illustrated in Fig. 6.

To derive the gross energy generation from methane generated from anaerobically digesting Welsh LIHD press fluid the following equation was used: (*T*_DM_ (39.8*vp_T_*))0.2778)0.85 = *x*_kWh_. Abbreviations are: *T*_DM_ = total LIHD biomass available in Wales per year (tonne DM); 39.8 the calorific value of methane (MJ/m^3^); *v* *=* CH_4_ production via anaerobic digestion (m^3^/t of press fluid); *p_T_* *=* press fluid production per tonne (concentrated); 0.2278 = conversion factor for MJ to kWh; 0.85 = conversion co-efficient for converting methane in an 85% efficient CHP unit; *x*_kWh_ = gross annual power output from converting methane from LIHD biomass from Wales (kWh per annum).

Gross energy generation from the combustion of Welsh LIHD derived pyrolysis oil was calculated using the following equation: ((*oil*_yield_/*T*_DM_)*E°*_OLHV_)0.2778 = *y*_kWh_. Abbreviations are: *oil*_yield_ = oil yield kg/tonne of dry matter following fast pyrolysis; *T*_DM_ = total LIHD biomass available in Wales per year (tonne DM); *E°*_OLHV_ = the mean lower heating value of fast pyrolysis oil made from LIHD biomass; 0.2278 = conversion factor for MJ to kWh; *y*_kWh_ = gross power output potential of fast pyrolysis oil via combustion (kWh per annum).

Gross energy generation from the combustion of Welsh LIHD derived char from fast pyrolysis was calculated using the following equation: ((*C*_yield_/*T*_DM_)*E°*_CLHV_)0.2778 = *Z*_kWh_. Abbreviations are: *C*_yield_ *=* char yield kg/tonne of LIHD dry matter following fast pyrolysis; *T*_DM_ = total LIHD biomass available in Wales per year (tonne DM); *E°*_CLHV_ = the mean lower heating value of char made by fast pyrolysis of Welsh LIHD biomass. *Z*_kWh_ = annual power production from the combustion of char derived from fast pyrolysis of Welsh LIHD biomass.

## 3 Results & discussion

**Fig. 3:**
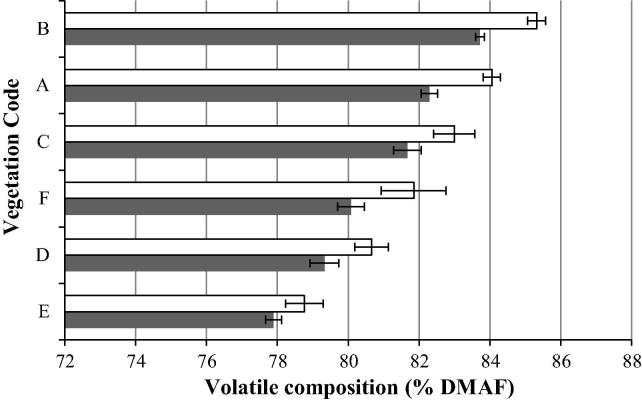
The impact of vegetation community and biomass processing on the mean volatile composition of biomass, silage (grey) and press cake (white). Silage = ensiled biomass; press cake = silage that has been hydrothermally pre-treated and mechanically dehydrated (HPMD). DMAF = dry matter ash free. For details of vegetation communities corresponding to codes A–F see Table 1.

There was a significant difference in the volatile contents of the silage and press cake across the six vegetation communities examined in the current study (*p* = <0.001). It is established that the volatile compositions established through TGA are positively correlated with the oil production capacity of a feedstock subjected to thermochemical conversion [37], [38]. It is therefore deemed a suitable parameter for distinguishing those samples most suited to experimental fast pyrolysis. On this basis biomass from the two sites with the most distinctive volatile compositions were chosen for thermochemical conversion. This provided as broad a product range as possible, given the capacity constraints on the subsequent fast pyrolysis work. Following sample screening using TGA it was established that the rush dominant feedstock had the highest volatile content compared with the other biomass types within the sample range and the bracken dominant feedstock had the lowest volatile content in the sample range (Fig. 3).

Statistical analysis showed the rush and bracken volatile compositions were the most distinct in the sample set, with both at the extremes of the established range (Fig. 3: B and E). The rush silage had a volatile composition of 84% and the rush press cake was 85% dry matter ash free (DMAF) volatiles. The mean bracken silage volatile composition was 78% and the mean press cake volatile composition was 79% DMAF. Rush and bracken were therefore selected for conversion via fast pyrolysis because they represented the most distinct volatile compositions within the dataset.

Further characterisation of the selected samples showed that press cake had the lowest ash and the lowest fixed C composition compared with silage, which explained the higher volatile composition. The fibrous composition of the press cake was generally lower than that of the silage (Table 3; cell wall polymers were partly dissociated through pressing); and thus the work confirms the effectiveness of the screw press for biorefining [4], [39] whereby the fibrous cell walls can be partly fractionated in green feedstocks.

There were relatively high concentrations of metals in the rush and bracken feedstocks but these were reduced by the pre-treatment process, as occurs for other feedstocks [28]. Pre-treatment of biomass by the use of a warm water wash and water removal by use of a screwpress, resulted in, on average, marginal increases (to 1.7–5.9% of original) in C, HHV and LHV, and decreases (to 8–45% of original) in concentrations of N, P, Ca and ash, and larger decreases (to 56–82% of original) in K, Mg and Na (Table 3). Most notably therefore the largest reductions were in the concentrations of the alkali metals K (81% reduction in rush press cake and 67% reduction in bracken press cake) and Na (82% reduction in rush press cake and 76% reduction in bracken press cake) and the alkaline earth metal Mg (66% reduction in rush press cake and 56% reduction in bracken press cake).

**Fig. 4:**
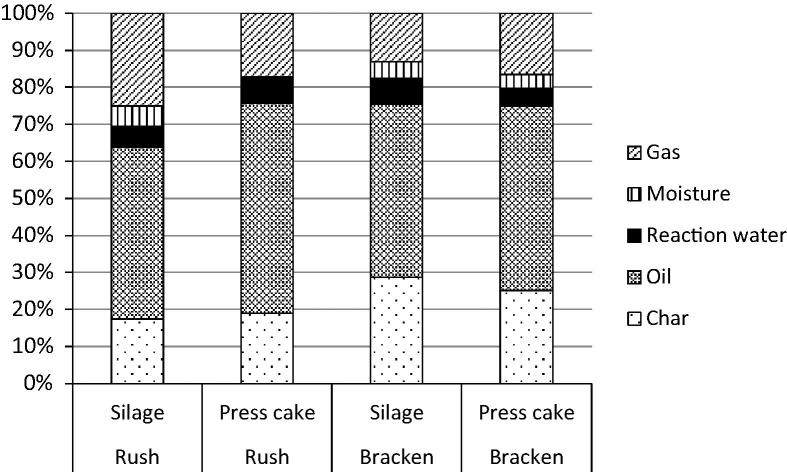
Product yields (% by mass) following fast pyrolysis of rush and bracken dominated feedstocks, following ensiling (Silage) and as a press cake once the silage was processed by being washed and pressed. Before processing the feedstocks were oven dried.

The observed demineralisation commonly occurs following HPMD [28], [29]. Indeed a primary role of HPMD in many processes is de-mineralisation for improved combustion fuel generation in conjunction with the production of a press fluid that can be used as a feedstock for anaerobic digestion. Once optimised the anaerobic digestion of the press fluids provides enough energy to drive the pressing procedure with excess power remaining: power that could be utilised for drying and milling biomass prior to fast pyrolysis [40]. So, irrespective of the impact of HPMD upon fast pyrolysis the pressing procedure has value because of the energy generated by anaerobic digestion of the resultant press fluid. According to this work the fibrous products remaining after pressing could potentially be optimised fast pyrolysis feedstocks, thereby integrating biological and thermochemical conversion into a unified process (Fig. 6). In support of this process option we found that partial demineralization of LIHD biomass through washing and pressing was associated with higher oil yields following fast pyrolysis (Fig. 4).

The HPMD of rush silage acted to increase oil yields following fast pyrolysis by 9%. Whilst bracken oil yields increased following HPMD, the difference was smaller (1.6%). These oil yields were within the ranges established following fast pyrolysis of the purpose grown biofuels willow and miscanthus [36].

In previous work a drop in mineral composition following HPMD had been beneficial because the fibrous product (employed in that case as a combustion fuel) had an improved emissions profile and a raised ash softening temperature [39]. During fast pyrolysis a reduction in the mineral composition has other benefits. Minerals can play a catalytic role in char formation according to Raveendran et al. [40] at the expense of oil production. Thus HPMD could theoretically decrease char production by reducing the catalysing mineral composition of the feedstock, increasing the percentage oil yield reflecting the work of Fahmi et al. [38] in which a negative correlation between ash (mineral) and bio oil yield was identified.

High ash content in the feedstock is associated with high reaction water concentrations and heavy organics in the product liquid [41]. This is due to secondary cracking where pyrolysis vapours are further degraded. The oil with the highest composition of reaction water was derived from the silage sample from the Bracken site (Fig. 4). This is the feedstock with the highest ash content indicating that secondary cracking may have taken place as a consequence. Secondary cracking is the breakdown of large molecules into smaller molecules; reaction water can be produced as a result and minerals catalyse these reactions.

The HPMD procedure increased bulk density by an average of 8% across both the rush and bracken dominated feedstocks examined in the current study. The rush derived silage and press cake had a much higher bulk density than the equivalent feedstock from bracken. The press cake samples had higher bulk densities compared to the silage samples. Rush press cake may therefore be the most cost-effective for transport compared to the bracken feedstocks.

The calorific values of the oils and chars produced in the current study were highest in those derived from rush (Table 4). With respect to the char heating values this variation possibly relates to the comparatively higher C composition of the rush chars and that reflected the feedstock compositions.

There appeared to be elevated CO_2_ production during fast pyrolysis of the bracken derived feedstocks (Table 5). Hydrogen gas and methane appear to be responsible for the elevated gas component in the mass balance (Table 6) relating to the rush silage feedstock run. The silage samples generate more hydrogen than the press cake samples under fast pyrolysis and the CO_2_ production was comparatively high during fast pyrolysis of the bracken derived feedstocks.

The chemical components of the pyrolysis oil that were predicted with a confidence of >70% (using the NIST spectral database) are shown in Table 7. The reduction in Amine composition in the oil produced using a rush press cake feedstock (following pressing) is particularly notable and does not follow the pattern of overall N composition, so the amines are not necessarily the end point of the N containing compounds in the oil fraction.

When more than twenty chemical species were identified at this level of confidence then the most abundant twenty species were tabulated based upon the area % of the total ion chromatogram. All of the GC–MS derived compositions of the oil fractions showed an abundance of levoglucosan, silane derivatives and organic acids (Table 7). Levoglucosan is a primary intermediate in thermal degradation and a common constituent of pyrolysis oil. Its production can be suppressed (with a simultaneous increase in char production) by using sodium chloride as a catalyst. Levoglucosan can be hydrolysed to glucose and this may provide a feedstock for anaerobic digestion or aerobic fermentation to produce methane or bioethanol respectively from lignocellulosic resources. This creates further potential options within an integrated bio-refinery process that could improve the overall economics of biomass refining [1]. However in the following section a different system is used to get a gross perspective on the potential.

This work expands the work done on the IFBB procedure by Wachendorf et al. [4] and optimized in the Kade system by Corton et al. [29], [39]. However, instead of pressing biomass to produce a solid fuel and a press fluid this system involves pressing biomass to produce a press fluid and a fast pyrolysis feedstock. This is a system that combines thermochemical and biological conversion routes (here on entitled Combi 1). Combi 1 may benefit the energy balance and feasibility of fast pyrolysis by creating secondary energy carriers. This may be achieved through char combustion and methane production and conversion via a combined heat and power unit (CHP). Those secondary power outputs can potentially be fed back into the system and contribute to the process energy requirements or utilised as energy carriers independently. It is also the case that Corton et. al. [29] optimized the IFBB press fluid digestion procedure by not adding water to the biomass before pressing. Though demineralisation was reduced a highly concentrated AD feedstock was generated. As biomass needs to be dried before fast pyrolysis (ideally) an implementation of the pressing procedure without washing would reduce drying costs significantly (through partially dewatering the biomass) whilst simultaneously producing an energy gain (via AD). As shown by Corton et al. [29] the energy generated from methane produced following the AD of the press fluid more than compensates for the energy required for pressing (14 kWh t^−1^ of fresh matter silage).

If a HPMD pre-treatment is implemented using LIHD feedstock, in an energy system incorporating fast pyrolysis of the resultant press cake then three energy carriers are produced. The press fluid can be subjected to anaerobic digestion [29]. The char generated during fast pyrolysis can be subjected to combustion providing an energy credit [42] and the oil can be upgraded to replace diesel fuel use in transport [41] or used in industrial burners with no upgrading. It is envisaged that in such a system (Fig 6) that the biogas and char combustion fuel would be utilised for power generation in order to benefit the systems energy and greenhouse gas (GHG) balance. A full life cycle analysis of the system is required in order to appreciate fully the implications for reducing GHG emissions. Here we present a potential system for further work and discussion and present only gross energy production potentials.

What is the potential size of the LIHD biomass resource? One case study examining the availability in Wales alone found that approximately 1 million tonnes of LIHD feedstock may be available annually [3] (not including un-harvestable areas or roadside verges).

There are many developing routes for bio-oil valorisation including co-firing in commercial boilers/burners, upgrading to transport fuels or biorefining for multiple outputs [43]. In Table 8 gross estimations are presented with regards to the potential of using the oil and char from Welsh LIHD biomass for commercial burner/boiler applications as reviewed by Czernik et al. (2004) [20]. Table 8 also shows the gross power production potential from digesting the press fluid [29].

It may be possible to dewater the digestate generated during AD and use it as a feedstock for slow pyrolysis as reported by Monlau [44]. Although the very high moisture composition of a press fluid digestate may make dewatering energetically challenging.

**Fig. 5:**
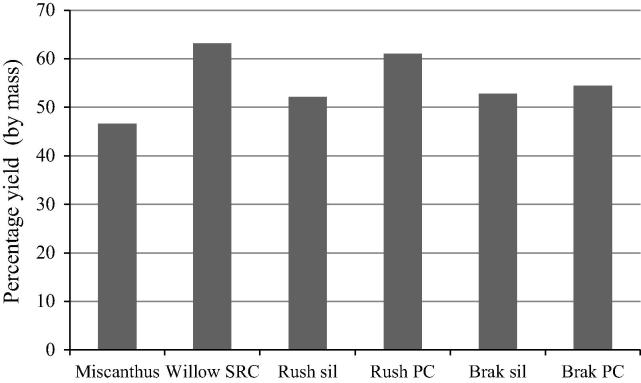
Total bio-oil yields generated from fast pyrolysis. LIHD biomass silage (sil) and warm water washed and pressed silage to form a press cake (PC) compared to yields of miscanthus and willow (short rotation coppice: SRC) determined using the same methods in the same laboratory [36].

**Fig. 6:**
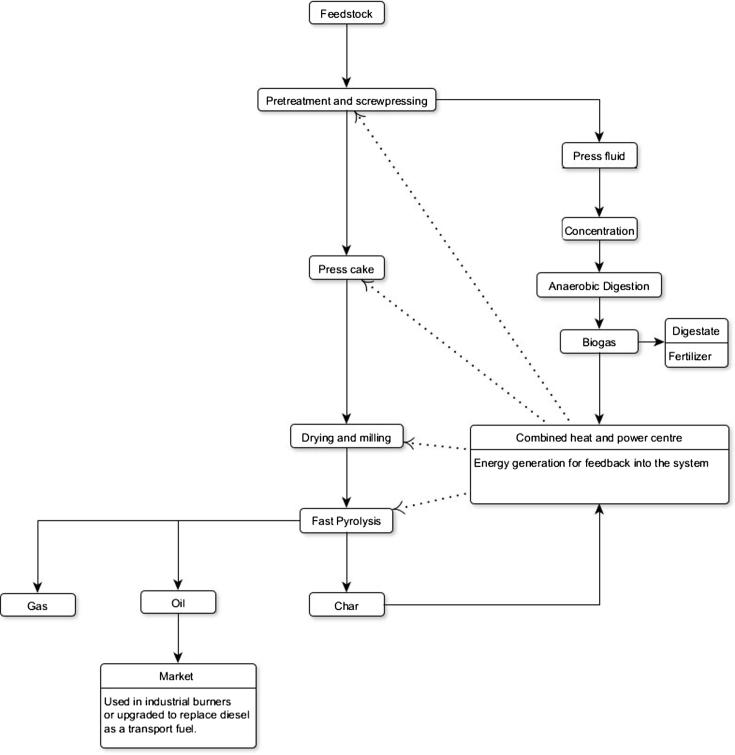
A potential system (Combi 1) for enhanced fast pyrolysis efficiency that utilises LIHD biomass in a system with multiple energy streams with feedback to support the primary conversion route (fast pyrolysis).

This work illustrates that LIHD biomass can be used to produce oil yields broadly in line with purpose grown energy crops (Fig. 5) following fast pyrolysis and is a novel feedstock for oil production via that process. The figures in Table 8 illustrate the potential scale of this system. If the power potential of the char and oil generated were to displace light oil (No. 2) the 3.9 × 10^5^ tonnes would be displaced (with a No. 2 oil having a LHV of 43 MJ/kg [45]. Following the developments up to date of the Combi 1 system a full LCA is now required in order to calculate meaningful net energy data, GHG balance and pollution potential. This will provide a more accurate picture as to the practical implications and the potential for Combi 1 to reduce GHG’s as well as benefitting biodiversity management.

## 4 Conclusion

The rush and bracken dominated feedstocks in this study were subjected to fast pyrolysis and the yields and properties were broadly in line with other biomass feedstocks, principally the dedicated energy crops miscanthus and willow. This was the case whether a HPMD pre-treatment had been applied or not. Following fast pyrolysis, rush PC generated a higher percentage yield of oil when compared to other feedstocks in the current work and comparable to the oil yields of willow (Fig. 5) using the same methods in the same laboratory [36]. The bracken oil yields were within the published range between willow and miscanthus (Fig. 5).

The growing of cultivated biofuels raises a number of issues. Purpose grown bioenergy crops can compete with food and feed crops for land cover. In addition land use change is a huge burden to biofuel production because the CO_2_ emitted in the process creates a carbon debt that needs to be repaid by production [46]. The use of LIHD biomass avoids the carbon costs of irrigation, fertilizer and pesticide production and application [47]. Conditional upon the species and the site, there can be a risk of unintentionally introducing invasive biofuel species when cultivating purpose grown biomass [48]. Conversely the management of conservation areas is associated with managing biodiversity, conserving ecosystem services and sequestering carbon. This work shows that in the case of fast pyrolysis, LIHD biomass can produce oil yields comparable to purpose grown willow and miscanthus whilst avoiding the aforementioned negative impacts. It is also notable that LIHD biomass is a novel feedstock for fast pyrolysis.

The LIHD biomass annual biomass yields are low compared to willow and miscanthus (3.21 t DM h^−1^ yr^−1^ [3]). However, unlike willow and miscanthus there is no competition with other land uses and there is already an estimated harvestable land area of LIHD biomass of 351,000 ha in Wales alone, and an estimated yield availability of 1 million t DM yr^−1^ (inclusive of a 7% estimated harvesting loss) [3]. Low input high diversity biomass represents an abundant bioenergy feedstock and currently an underutilised natural resource arising from the management of semi-natural habitats and landscapes. These habitats are purposefully kept at low fertility levels to encourage biodiversity so removing biomass and the associated minerals is not considered to be an issue and is practiced regularly across the UK. It is worth pointing out that the deposition of atmospheric N on upland areas is problematic for plant diversity, especially in areas adjacent to urban centres and a cutting management could potentially be one way of denitrifying these areas. It is also evident that LIHD biomass in the UK grows in localities that are not attached to national gas grids; oil burning systems are prevalent in such areas; this is a favoured route to valorisation for pyrolysis oil [49].

This paper presents the conversion of LIHD biomass via fast pyrolysis. This is an original combination of feedstock to conversion route, and in combination with the proposed system represents a new perspective that integrates thermochemical and biological conversion into one unified process to benefit the energy balance. Estimates of the gross energy output indicate that significant energy generation may be possible with this system utilising the oil and char in industrial burners. Clearly a detailed life cycle assessment is required to generate substantial energy output data. Preliminary data suggests that annually in Wales (UK) the system could displace 3.9 × 10^5^ tonnes of No. 2 Light Fuel Oil, potentially contributing to significant GHG reductions for industry.

## Figures and Tables

**Table 1:** The broad habitat types, dominant plant species and abbreviations used to represent each of the six vegetation communities used in the current study. The abundance of the primary dominant species is expressed as % cover.

Vegetation code	Broad habitat	Dominant species
A	Neutral grassland	*Molinia caerulea* (60%), *Agrostis canina* (25%), *Juncus acutiflorus* (22%).
B	Fen, marsh, swamp	*Juncus effuses* (51%), *Molinia caerulea* (19%), *Deschampisia cespitosa* (5%).
C	Acid grassland	*Juncus effuses* (35%), *Agrostis canina* (28%), *Carex echinata* (27%).
D	Acid grassland	*Vaccinium myrtillus* (44%), *Nardus stricta* (7%), *Deschampsia cespitosa* (32%).
E	Dense bracken	*Pteridium aquilinum* (57%), *Agrostis capillaris* (37%).
F	Acid grassland	*Molinia caerulea* (32%), *Nardus stricta* (26%), *Festuca ovina* (40%).

**Table 2:** The run parameters used during bench scale experimental pyrolysis.

Parameter	Detail
Pyrolysis temperature	500 °C
Feed rate	3 g min^−1^
Electrostatic precipitator	15 kV, 0.5 mA
Condensing medium	Water, dry ice and acetone
Reactor type	Fluidised bed
Fluidising medium	150 g quartz sand, particle size between 500–600 μm
Flush gas	Nitrogen
Fluidising medium flow rate	12 l/m^3^
Feed-stock particle size	500–600

**Table 3:** Biomass characteristics and fast pyrolysis product yields of LIHD biomass compared to values for miscanthus and willow (short rotation coppice: SRC) determined using the same methods in the same laboratory [36]. LHV = lower heating value (MJ/kg^−1^); HHV = higher heating value (MJ/kg^−1^); Bulk *D* = bulk density (kg/m^3^); ADL = acid detergent lignin (% d.b.); VM = volatile matter (% d.b.); FC = fixed carbon (% d.b.); Hemi = hemicellulose (% d.b.); TCW = total cell wall (% d.b.); Oil org = oil organics; RW = reaction water; d.b. = dry matter basis. Elemental compositions are presented as mean (*n* = 3) % d.b.

	Miscanthus	Willow SRC	Rush sil	Rush PC	Brak sil	Brak PC
C	46.95	48.48	45.6	46.63	42.51	45.03
H	5.85	5.74	6.05	5.86	5.35	5.64
N	0.92	1.87	1.51	1.14	2.09	1.92
O	46.28	43.91	43.31	43.43	41.04	42.01
Moisture (%)	4.55	5.71	5.03	4.28	5.14	3.56
VM	75.62	81.19	83.72	85.32	78.76	77.9
FC	19.92	15.85	16.28	14.68	21.24	22.1
Ash	4.46	2.96	3.46	2.93	8.6	5.4
HHV	18.38	19.06	18.26	18.58	17.24	18.03
LHV	17.1	17.81	16.94	17.29	15.97	16.8
Bulk D	–	–	254	261	174	202
Ca	0.18	1.15	0.34	0.21	0.45	0.32
K	1.2	0.59	0.48	0.09	0.92	0.3
Mg	0.15	0.16	0.12	0.04	0.25	0.11
Na	–	0.01	0.17	0.03	0.17	0.04
P	0.07	0.19	0.11	0.06	0.2	0.15
Cellulose	52.13	49.3	34.25	27.85	33.86	25.15
Hemi	25.7	14.1	36.89	29.39	25.82	31.36
Lignin	12.5	20	7.06	7.09	16.64	6.29
TCW	90.33	83.4	78.2	64.33	76.32	62.8

Fast pyrolysis yields (% by mass)						
Char total	31.37	14.43	19.67	20.45	32.41	27.52
Bio-oil	46.61	63.17	52.14	61.03	52.79	54.43
Oil Org.	40.53	55.47	39.63	53.47	39.86	45.14
Oil RW	6.08	7.7	6.23	7.51	7.86	5.26
Gas total	9.13	13.03	28.19	18.52	14.81	18.06
Closure	87.11	90.63	90.08	95.45	91.99	99.31

**Table 4:** Elemental compositions (% dry matter basis) and heating values of fast pyrolysis oils from rush and bracken dominant feedstocks, following ensiling to make silage and also subsequent processing (a warm water wash and passing through a screw press) to form a press cake. The Figures are means from duplicate analyses. Sulphur concentrations were below the detection level. LHV = lower heating value; HHV = higher heating value.

Feed-stock origin	Pre-treatment	C	H	N	S	O	HHV (MJ/kg^−1^)	LHV (MJ/kg^−1^)
Rush dominant	Silage	50.0	8.0	1.0	0	41.0	20.6	13.0
Rush dominant	Press cake	50.0	8.0	2.3	0	39.8	20.8	15.3
Bracken dominant	Silage	49.6	7.1	2.2	0	41.1	20.3	12.3
Bracken dominant	Press cake	48.5	7.5	2.7	0	41.3	19.9	12.3

**Table 5:** Elemental compositions (% dry matter basis), mineral compositions, higher heating values (HHV; MJ/kg^−1^) and lower heating values (LHV; MJ/kg^−1^) of the chars made from fast pyrolysis of rush and bracken dominant feedstocks, following ensiling to make silage and also subsequent processing (a warm water wash and passing through a screw press) to form a press cake. The Figures are means from duplicate analyses.

Feedstock	Pre-treatment	C	H	N	Ca	K	Mg	Na	P	O	HHV	LHV
Rush dominant	Silage	60.6	3.0	1.9	1.1	1.5	0.4	0.5	0.3	31.7	22.4	21.8
Rush dominant	Press cake	59.8	3.3	1.9	0.8	0.3	0.2	0.1	0.2	33.3	22.4	21.7
Bracken dominant	Silage	56.5	2.7	1.7	1.1	2.0	0.6	0.3	0.5	36.1	20.9	20.3
Bracken dominant	Press cake	60.8	2.8	1.7	0.8	0.6	0.3	0.1	0.4	30.8	22.3	21.7

**Table 6:** Composition of the non-condensable gaseous fractions obtained from fast pyrolysis of rush and bracken dominant feedstocks, following ensiling to make silage and also subsequent processing (a warm water wash and passing through a screw press) to form a press cake. The figures represent percentage composition by mass within the gaseous product stream.

Gaseous component	Site	Rush dominant	Rush dominant	Bracken dominant	Bracken dominant
	Pre-treatment	Silage	Press cake	Silage	Press cake
H_2_		0.64	0.32	0.54	0.33
CO		30.29	25.38	19.65	23.26
Methane		4.58	3.73	3.38	3.82
CO_2_		28.17	27.27	29.57	28.74
Ethene		5.07	5.62	5.33	5.59
Ethane		7.48	5.94	8.58	6.04
Propene		6.60	8.26	8.44	8.36
Propane		8.16	11.39	11.82	11.52
n-Butane		9.01	12.15	12.76	12.24

**Table 7:** Compounds identified in the oil fraction from GC–MS analysis of fast pyrolysis of rush and bracken dominant feedstocks, following ensiling to make silage and also subsequent processing (a warm water wash and passing through a screw press) to form a press cake. The figures represent percentage composition by mass within the oil stream.

Component	Plots & pre treatment			
	Rush dominant	Rush dominant	Bracken dominant	Bracken dominant
	Silage	Press cake	Silage	Press cake
Alcohol	2.38	3.65	3.4	5.74
Aldehyde	0	0	0	2.44
Alkane	4.5	2.64	3.47	3.63
Alkene	0	0	2.12	0
Amine	3.65	0	0	5.78
Aromatic hydrocarbon	0.54	0.215	1.15	0
Carboxylic acid	0	2.75	0	2.24
Lactate	0	0	5.57	1.79
Ether	8.83	7.5	5.65	6
Ketone	0	0	2.69	2.26
Levoglucosan	8.94	21.98	6.96	8.98
Organic acid	15.43	22.86	28.39	17.36

**Table 8:** Gross energy output potential of the energy carriers generated from the Combi 1 system (methane, oil and char) when LIHD biomass from Wales is used as a feedstock.

Energy carrier	Amount generated per year	Conversion route	Gross power production (MW h/annum)
Press fluid (post concentration)	3.29 × 10^5^ (m^3^)	Anaerobic digestion	8.1 × 10^3^
Char	2.5 × 10^5^ (tonne)	Combustion	1.5 × 10^6^
Oil	6.2 × 10^5^ (tonne)	Combustion	2.3 × 10^6^

Total gross energy output			3.808 × 10^6^
